# Phase II randomized, double-blind, placebo-controlled study of whole-brain irradiation with concomitant chloroquine for brain metastases

**DOI:** 10.1186/1748-717X-8-209

**Published:** 2013-09-08

**Authors:** Luis L Rojas-Puentes, Marcelino Gonzalez-Pinedo, Alejando Crismatt, Alette Ortega-Gomez, Carlos Gamboa-Vignolle, Rodrigo Nuñez-Gomez, Yusmiren Dorantes-Gallareta, Claudia Arce-Salinas, Oscar Arrieta

**Affiliations:** 1 Medical Oncology Department, Instituto Nacional de Cancerología de México(INCan), San Fernando N22 Colonia Sección XVI, Tlalpan Mexico City, Mexico; 2 Translational Medicine Laboratory, INCan, Mexico City, Mexico; 3 Radiooncology Department, INCan, Mexico City, Mexico; 4 Head and Neck Unit, INCan, Mexico City, Mexico; 5 Thoracic Oncology Clinic, INCan, Mexico City, Mexico; 6 Experimental Oncology Laboratory, INCan, Mexico City, Mexico

**Keywords:** Brain metastases, Radiation therapy, Chloroquine, Whole-brain radiation, Radiosensibilization

## Abstract

**Background and purpose:**

Chloroquine (CLQ), an antimalarial drug, has a lysosomotropic effect associated with increased radiationsensibility, which is mediated by the leakage of hydrolytic enzymes, increased apoptosis, autophagy and increased oxidative stress *in vitro*. In this phase II study, we evaluated the efficacy and safety of radiosensibilization using CLQ concomitant with 30 Gray (Gy) of whole-brain irradiation (WBI) to treat patients with brain metastases (BM) from solid tumors.

**Methods:**

Seventy-three eligible patients were randomized. Thirty-nine patients received WBI (30 Gy in 10 fractions over 2 weeks) concomitant with 150 mg of CLQ for 4 weeks (the CLQ arm). Thirty-four patients received the same schedule of WBI concomitant with a placebo for 4 weeks (the control arm). All the patients were evaluated for quality of life (QoL) using the EORTC Quality of Life (QoL) Questionnaire (EORTC QLQ-C30) (Mexican version) before beginning radiotherapy and one month later.

**Results:**

The overall response rate (ORR) was 54% for the CLQ arm and 55% for the control arm (p=0.92). The progression-free survival of brain metastases (BMPFS) rates at one year were 83.9% (95% CI 69.4-98.4) for the CLQ arm and 55.1% (95% CI 33.6-77.6) for the control arm. Treatment with CLQ was independently associated with increased BMPFS (RR 0.31,95% CI [0.1-0.9], p=0.046).The only factor that was independently associated with increased overall survival (OS) was the presence of< 4 brain metastases (RR 1.9, 95% CI [1.12-3.3], p=0.017). WBI was associated with improvements in cognitive and emotional function but also with worsened nausea in both patients groups. No differences in QoL or toxicity were found between the study arms.

**Conclusion:**

Treatment with CLQ plus WBI improved the control of BM (compared with the control arm) with no increase in toxicity; however, CLQ did not improve the RR or OS. A phase III clinical trial is warranted to confirm these findings.

## Introduction

Brain metastases (BM) are the major neurological complication from cancer [1-3]. As many as 40% of adult patients with disseminated cancer experience brain metastases [4]. The two major primary malignancies associated with BM are lung and breast cancer [2,5]. With supportive care and corticosteroid therapy, the median survival of cancer patients with BM is approximately 1-2 months [6]. There have been many attempts to improve this outcome, including the treatment of brain metastases with whole brain irradiation (WBI) surgery, stereotactic radiosurgery or a combination of these methods.The result has been a median survival time that ranges from 6.5 to 10 months [7,8].Different primary tumor control treatment strategies could improve the OS of these patients [9,10]. Combinations of WBI and several chemotherapeutic agents, including halogen pyrimidines, fluoropyrimidines, gemcitabine and platinum compounds, have failed to produce the expected therapeutic benefits [11]. There have been inconclusive benefits from the use of temozolomide as a radiosensitizer [12-14].

When used as an antimalarial drug, chloroquine (CLQ) can cause alterations in cell function, affecting lysosomal membranes (lysosomotropic action) and producing DNA damage [15,16]. The lysosomotropic effect increases the cancer sensitivity to radiation and recruits diverse antitumor mechanisms that include p53-dependent apoptotic activation and the inhibition of autophagic protein degradation [17]. In tumoral cells, CLQ increases oxidative stress, lysosomal accumulation, mitochondrial depolarization and caspase activation [18]. There is evidence that CLQ may also increase the oxidative stress induced by radiotherapy [19]. *In vitro* studies performed in glioma cells have demonstrated that the anticancer efficacy of CLQ is mediated by the induction of apoptosis and the inhibition of autophagy. A murine melanoma model exhibited caloric restriction and inhibition of autophagy after CLQ treatment [17,18]. Clinical studies conducted in primary brain tumors have suggested that CLQ may improve OS when CLQ is administered in addition to conventional therapy or as an adjuvant to conventional surgery, chemotherapy or radiosurgery [20,21].

The aim of this study was to evaluate the radiosensitizing effect of CLQ combined with WBI in patients with brain metastases from solid tumors.

## Patients and methods

We conducted a prospective, double-blind, randomized, phase II clinical trial at a single institution (Instituto Nacional de Cancerolgía, México City).The Institutional Ethics and Scientific Committees approved the study protocol (008-033-OMI) (CD-449-08), and the study was conducted according to the Declaration of Helsinki and was registered in ClinicalTrials.gov (NCT01894633). All the patients provided fully informed consent to participate. The primary objective was overall response rate (ORR) in brain metastases. The secondary objectives comprised toxicity, the progression free survival of brain metastases (BMPFS), overall survival (OS), event-free survival (EFS) (BMPFS or death) and quality of life.

### Patients

Eligible patients were 18-80 years of age, had at least one BM ≥ 1 cm upon MRI analysis, had a Karnofsky performance status (KPS) ≥70 and were classified as RTOG-RPA I or II. The laboratory requirements were an absolute neutrophil count of >1500/mm^3^,a platelet count ≥ 100,000/mm^3^,blood urea nitrogen ≤25 mg/dL,serum creatinine ≤ 1.5 mg/dL,serum bilirubin ≤ 1.5 ml/dL and alanine aminotransferase and aspartate aminotransferase levels≤ 2 times the upper normal limit. Patients who were eligible for radiosurgery or stereotactic radiotherapy (patients with three or less brain metastases, size up to 4 cm and primary tumor controlled) or who had a history of previous brain radiotherapy were excluded. The clinical evaluations included a complete clinical history, a KPS assessment and physical and neurological examinations.

### Study design

Patients were randomly assigned (1:1) without stratified by a random number table to receive CLQ plus WBI (CLQ arm) or a placebo plus WBI (control arm). The investigators and patients were blinded for treatment assigned groups (double blind) The patients in the CLQ arm received 30 Gy of total brain radiotherapy in 10 daily fractions from Monday to Friday. Furthermore, the CLQ arm received a daily single dose of 150 mg CLQ po1 hour prior to the radiation treatment, beginning during the first radiotherapy fraction and continuing for 28 days. WBI was applied with two parallel and opposing fields using a 1.25- or 6-Mv photon beam. The dose was calculated in the midplane along the central axis. The patients in the control arm received 30 Gy of whole-brain irradiation in 10 daily fractions and an oral matching placebo for 28 days. In addition, after the radiation treatment and as indicated, patients received the appropriate systemic treatment for their primary tumor and/or for non-brain metastases. Brain metastasis progression was treated with cranial re-irradiation or radiosurgery whenever possible.

### Follow-up

The patients were evaluated every week during treatment and then every month until they were lost to follow-up or died. During treatment, the patients underwent clinical evaluations and biochemical profiling. After three months of follow-up, the patients were evaluated using brain MRIs. When the patients presented neurological symptoms prior to 3 months were evaluated by MRI and considered as patients with brain disease progression. Patients without neurological symptoms and who died prior to getting MRI, they did not register as brain diseases progression. A radiologist from our institution evaluated the brain MRIs in accordance with the RECIST 1.1 criteria [22]. The radiologist performed the evaluation in a blinded fashion. The overall response (ORR) encompassed complete responses (CRs) and partial responses (PRs).Non-response included stable disease (SD) and progressive disease (PD). Adverse events were evaluated every week during treatment (28 days) and were graded according to the NCI Common Terminology Criteria (NCI-CTCAE v3.0).The local Ethics Committee recommended ophthalmologic evaluations due to the potential side effects of CLQ; therefore, all the patients were evaluated for visual accuracy and underwent two fundus examinations by a certified ophthalmologist: one after the CLQ treatment ended and another two months later.

The 30-item EORTC Quality of Life (QoL) Questionnaire (EORTC QLQ-C30) version 3.0 (Mexican version) was used in this trial [23]. The EORTC QLQ-C30 v3 consisted of five multi-item functional scales, three symptom scales, a global health status/QoL scale, and six single items. The scores were transformed according to the instructions in the EORTC QLQ-C30 v3 manual. The possible scores on all the scales and single items ranged from 0 to 100 points. Higher scores on the functional and global health status QoL scales reflect better functioning. On the symptom scales, higher scores indicate larger numbers of symptoms or problems. The QoL questionnaires were completed 1 day before radiotherapy began and 1 month later.

### Statistical analysis

The sample size was calculated with a two-sided test using a type-I error probability of 0.05 and a power of 0.80. Thirty-five patients were required for each treatment arm to detect a difference of 0.30 in the ORR (according to the results of previous studies with temozolomide vs placebo) [12]. Intention-to-treat analyses were performed. The ORR and other categorical variables were estimated with the chi-squared test or Fisher’s exact test. Variables with significant or borderline significant values (p < 0.01) were included in a multivariate logistic regression analysis.

The BMPFS was measured from the treatment initiation until the date of brain’s disease progression (determined clinically or by brain MRI). The overall survival was measured on the date of death or the last follow-up visit. Event-free survival (EFS) was defined as the BMPFS or death, whichever occurred first. The survival variables were estimated using the Kaplan-Meier method, and the survival rates of the treatment arms were compared using the log-rank test; the Breslow test was applied if the two survival curves crossed. Adjustments for potential confounding variables were performed with a Cox proportional hazards regression model. QoL comparisons between the treatment arms were performed before and after radiotherapy and were analyzed using the Wilcoxon related samples test. Differences of≥10% on the EORTC QLQ-C30 scale were considered clinically significant. Statistical significance was determined as a two-sided p-value ≤0.05.

A priori, we expected the following clinical characteristics to have significant effects on treatment response: gender, age (<55 or ≥55 years, based on the patients media, performance status (Karnofsky Index), number of metastases to the CNS (<4 or ≥ 4 metastases), histology and the interval in months between the cancer diagnosis and the appearance of brain metastases (at diagnosis or during a recurrence). The outcomes are reported with 95% confidence intervals (CIs). The statistical calculations were performed with the SPSS version 17 software package (SPSS, Inc., Chicago, IL, USA).

## Results

**Figure 1:**
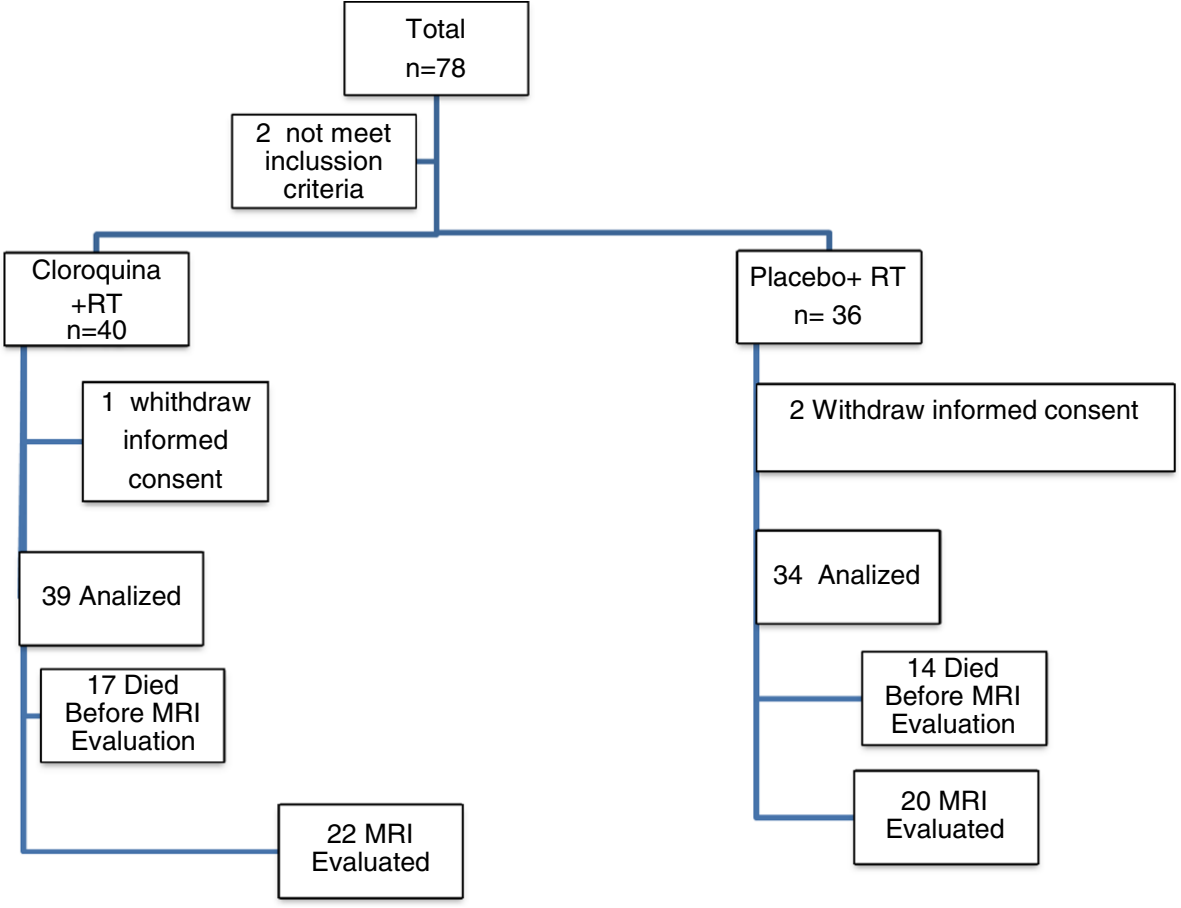
Consort.

From January 2011 to February 2012, 78 patients were evaluated for participation in this clinical trial. Two patients did not meet the inclusion criteria. Among the 76 patients who were enrolled in the study, 40 were randomly assigned to the CLQ arm and 36 were assigned to the control arm. Three patients withdrew their consent, including one in the CLQ arm two in the control arm. Ultimately, there were 39 patients in the CLQ arm 34 patients in the control arm. Seventeen patients in the CLQ arm and fourteen in the control arm died before the radiological evaluation. Only 22 patients from the CLQ arm and 20 patients from the control arm were evaluated with MRI during the third month after WBI. The follow-up period ended in February 2013. The CONSORT diagram for this study is presented in Figure 1.

The patients’ median age was 54 ±12 years. Among the patients, 53 (72.6%) were women and 20 (27.4%) were men. The two most common malignancies were lung and breast cancers (74% and 20.5%, respectively), and the other primary tumors observed were melanoma (2.7%), kidney cancer (1.4%) and primary unknown (1.4%). All the patients were in good PS, with a median Karnofsky index of 80%. Ninety-six percent of the patients were classified as RTOG-RPA II. The brain metastases were identified at the diagnosis of the primary tumor in the 57% of the cases and as a recurrence or progression in 42.5% of the patients. The median of number of metastases was three (ranges 1 to 30), 50 patients (68.5%) had ≤ 4 metastases; 12 patients (16.4%) had 5-8 metastases and 11 patients (15.1%) had ≥ 9 metastases. The median size was 22.8 ± 13 mm. The main characteristics of the patients are shown in Table 1. There were no significant differences between the arms in any of the above characteristics.

**Table 1:** Patient characteristics

**Characteristic**	**CLQ arm**	**Control arm**	**p**^§^
	**n = 39**	**n = 34**	
Gender			
Male	11(28.2%)	9 (14.7%)	
Female	28 (71.8%)	25 (85.3%)	0.868
Age (median)	55.7 ± 13	52 ± 10.6	
Range			
<55	18 (46.1)	21 (61.8.8%)	0.182
≥55	21 (53.91.8%)	13 (38.2.2%)	
KPS			
<80	8 (20.5%)	6 (17.6%)	0.756
≥80	31 (79.5%)	28 (82.4%)	
No. metastases			
<4	28 (71.8%)	22 (64.7%)	0.564
≥4	11 (28.2%)	12 (35.3%)	
Histology			
NSCLC and others	33 (84.6%)	25 (73.54%)	0.242
Breast cancer	6 (15.4%)	9 (26.5%)	
RPA			
I	1 (2.5%)	2 (5.9%)	0.476
II	38 (97.5%)	32 (94.1%)	
Time of brain metastasis			
During primary tumor diagnosis	23 (58.9%)	19 (55.9%)	0.816
Recurrence or progression	16 (41.151.6%)	15 (44.1%)	

*Abbreviations*: *CLQ* chloroquine, *KPS* Karnofsky performance score, *NSCLC* non-small-cell-lung cancer, *RPA* recursive partitioning analysis, *CHT* chemotherapy. ^§^Person Chi square test.

Complete response, partial response, stable disease and progression were observed in 4.9%, 48.8%, 43.9% and 2.4% of the patients, respectively. None of the studied clinical factors were associated with response. The relationship between the overall response rate (ORR) and the clinical factors are shown in Additional file 1. There were no differences in the ORR between the arms (54% for WBI plus CLQ vs. 55% for the control arm [ORR 1.08, 95% CI (0.3-3.7), p =0.92]). Table 2 shows the radiological response according to the RECIST criteria for both arms.

**Table 2:** Radiological response (RECIST criteria)

	**CLQ arm**		**Controlarm**		**p**
	**n=22**	**%**	**n=20**	**%**	
Complete	1	4.5	2	10	.915
Partial	11	50	9	47.3	.867
Stable disease	9	40.9	9	47.3	.678
Progressive disease	1	4.5	0	0	.347
Objective response	12	54	11	55	.902

*Abbreviations*: *CLQ + WBI* Cloroquine plus Whole-brain irradiation.

No toxicity (grade 4 or 5) was observed in either arm, and there were no significant differences in toxicity between the arms. The most frequent side effects reported in both arms were headache, dizziness, nausea, and vomiting. Additional file 2 lists the incidences of adverse events. The ophthalmologic follow-up revealed no evidence of visual side effects.

**Figure 2:**
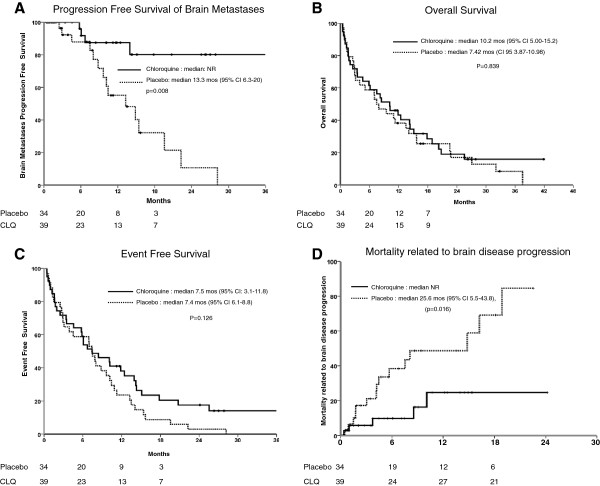
**Efficacy of Chloroquine. A.** Progression Free Survival of Brain Metastases. **B.** Overall Survival. **C.** Event Free Survival. **D.** Mortality related to brain disease progression.

The median follow-up time was 8.4 months (SD 9.4 months) for the overall study population. The median BMPFS was 22.3 months (95% CI 10.5-34). The CLQ arm did not reach the median BMPFS, whereas the BMPFS of the control arm was 13.3 months (95% CI 6.3-20, p=0.008) (Figure 2A). The BMPFS at one year was 83.9% (95% CI 69.4-98.4) for the CLQ group and 55.1% (95% CI 33.6-77.6) for the placebo group. The univariate analysis showed that age, primary tumor type and treatment were associated with BMPFS (Table 3). The multivariate analysis showed that CLQ treatment was the only factor significantly associated with better BMPFS (RR: 0.31, 95% CI 0.1-0.9, p=0.046).

**Table 3:** Factors associated with progression-free survival in patients with brain metastases.

**Factor**	**Median (months)**	**CI (95%)**	**Univariate analysis**	**RR**	**CI (95%)**	**Multivariate analysis**
			***P***^§^			**P**^§^
Gender						
Male	NA	(11.3-22.9)	0.257			
Female	19.6					
Age (years)						
<55	13.9	(8.8-19.1)	<0.001	0.22	(0.046 - 1.07)	0.06
≥55	NA					
KPS						
<80	28.1	(13.9 - 42)	0.946			
≥80	22.3	(12.3-32.3)				
Number of metastases						
<4	22.3	(11.3 - 33.2)	0.351			
≥4	14.9	(1.9 - 27.9)				
Histology						
NSCLC and others	NA	(6.6 -11.0)	<0.001	0.46	(0.13 – 1.7)	0.24
Breast cancer	8.8					
Time of brain metastasis						
During primary tumor diagnosis	22.3	(5.5 - 39)	0.089			
During recurrence	19.6	(10.1 - 29)				
Treatment						
Control arm	13.2	(6.3 - 20.1)	0.001	0.31	(0.1 – 0.9)	0.046
CLQ arm	NA					

*Abbreviations*: *SE* standard error, *KPS* Karnofsky performance status, *NSCLC* non-small-cell lung cancer, *CHT* chemotherapy, *CLQ* chloroquine. ^§^*P* log-rank test.

The median OS was 8.4 months (95% CI 4.8-12.1). There was no difference in the OS between the treatment arms. In the CLQ arm, the median OS was 10.2 months (95% CI5.00-15.2), whereas the control arm median OS was 7.42 months (CI 95 3.87-10.98, p= 0.839) (Figure 2B). The univariate analysis demonstrated that gender and the number of metastases were significantly associated with OS and showed a trend with PS. In the multivariate analysis, the only factor associated with increased OS was the number of metastases (< 4 vs.> 4 metastases) (RR 1.9,95% CI: 1.12 -3.3, p =0.017) (Table 4).

**Table 4:** Factors associated with overall survival in patients with brain metastases

**Factor**	**Median (months)**	**CI (95%)**	**Univariate analysis**	**RR**	**CI (95%)**	**Multivariate analysis**
			***p***^§^			**p**^§^
Gender						
Male	6.9	(5.9 -8.1)	0.05	0.65	(0.37 -1.3)	0.129
Female	11.3	(4.4 -18.1)				
Age						
<55	8.2	(4.3 -12.1)	0.44			
≥55	8.4	(3.5 -13.4)				
KPS						
<80	5.1	(0 - 10.4)	0.1	0.8	(0.48 -1.3)	0.38
≥80	12.4	(4.9 -19.9)				
Number of metastases						
<4	13.5	(9.6 -17.4)	0.002	1.9	(1.12 -3.3)	0.017
≥4	2.9	(1.1- 4.8)				
Histology						
NSCLC and others	7.9	(3.8 -11.9)	0.67			
Breast cancer	4.5	(1.9 -17)				
Time of brain metastasis						
During primary tumor diagnosis	10.2	(4.3-16)	0.743			
During recurrence	8.2	(3.9-12.5)				
Treatment						
Control arm	7.42	(3.8 -11)	0.637			
CLQ arm	10.1	(4.9 -15.3)				

*Abbreviations*: *SE* standard error, *KPS* Karnofsky performance status, *NSCLC* non-small-cell lung cancer, *CHT* chemotherapy, *CLQ* chloroquine. ^§^*P* log-rank test.

The median event (progression or death)-free survival was 7.5 months (95% CI 5.3-9.7). There was no difference between the CLQ arm (7.5 months; 95% CI: 3.1-11.8) and the control arm (7.4 months; 95% CI 6.1-8.8) (p =0.126) (Figure 2C). The only factor associated with EFS was the number of metastases. The median EFS was 10.1 months (95% CI 7.1-13.0) for the group with < 4 metastases, compared with 2.9 months (95% CI 1.1-4.8) for the group with ≥ 4 metastases (p=0.04) (Additional file 3). When we analyzed the death due to brain disease progressive only, the median was 27.1 months (95% CI 21.8-32.4). Patients of CLQ arm do not reach median and patients of control arm had a median of 24.6 months (95% CI 5.5-43.8), (p=0.016) Figure 2D.

The QoL scores of all the patients on the functional, emotional, cognitive and social scales exhibited clinically significant changes (10-point differences) before and after treatment with either CLQ or placebo. Only the cognitive function scale demonstrated a statistically significant change (p=0.05). There was also an increase in nausea after treatment (Additional file 4). There is no increase in adverse effects in CLQ arm compared with control arm. (Additional file 2). There were no differences in QoL between the treatment arms (Additional file 5).

## Discussion

Our results suggest that CLQ improves the local control and PFS of patients with BM when it is used concurrently with WBI, and there is no impact on the ORR or OS. This is the first study to evaluate CLQ for the treatment of BM; however, a previous study showed that CLQ treatment during radiation and chemotherapy improved the OS of patients with glioblastoma multiforme (GBM) compared with a placebo. In a prospective, controlled, randomized trial, 18 patients with GBM underwent standard treatment with surgery, chemotherapy, and radiotherapy. Nine patients received an additional 150-mg dose of chloroquine daily beginning 1 day after surgery and continuing through the observation period. Nine matched patients were included as controls. There was a significantly longer survival in the chloroquine-treated patients than in the controls (33 +/- 5 and 11 +/- 2 months, respectively [p < 0.0002]) [24]. The same authors published a randomized, double-blind, placebo-controlled trial in which 30 postsurgical patients were allocated to receive a150-mg daily dose of CLQ or placebo for twelve months beginning on the fifth day after surgery. All the patients also received conventional chemotherapy and radiotherapy. The median survival after surgery was 24 months for the CLQ-treated patients and 11 months for the placebo group. Although no statistically significant difference was observed, the rate of death among the patients receiving CLQ was approximately one-half that of the patients receiving the placebo (hazard ratio, 0.52 [95% CI, 0.21 to 1.26]; P = 0.139) [20].

There are some differences between our trial and the above studies, particularly in the duration of CLQ treatment. In our trial, CLQ was administered for four weeks; for the first two weeks it was combined with WBI, and for the next two weeks it was administered alone. In the Sotelo et al. study, CLQ was administered for one year and was combined with carmustine. This combination has a possible synergistic mechanism of action, with enhancement of the antimutagenic action; this could be the explanation for the better outcomes observed in that study [20,21]. In an *in vitro* study of glioma cells, Reyes et al. showed that a combination of carmustine and quinacrine (an antimalarial drug) increased the antineoplastic effect initially obtained with carmustine alone. In the long term, this combination led to a high percentage of tumor remissions compared with cells treated with carmustine alone (55 and 16%, respectively; p <0.01). The authors postulated that quinacrine might prevent carmustine resistance by reducing the extent of the primary DNA rearrangements that are responsible for the appearance of mutant clones [24,25]. In our study, it was not possible to continue the CLQ treatment for a longer time because all the patients required different chemotherapies specific for their primary tumors.

In contrast to GBM patients who die because of local progression, most of the patients with BM died from extracranial tumor progression. This finding may explain why improving the BMPFS did not impact the OS; nonetheless, the local control of BM has great relevance for quality of life.

Certain intracellular mechanisms may explain the effect of CLQ as a radiosensitizer. CLQ affects lysosomal membranes and activates several antitumoral actions, such as p53-mediated apoptosis, oxidative stress, caspase activation, and other actions that potentiate the toxic effects of radiation in tumoral cells [15-19]. In a serum-deprived U251 glioblastoma line, CLQ rapidly killed serum-starved cancer cells *in vitro* by an independent autophagy mechanism. CLQ induced lysosomal accumulation and oxidative stress, leading to mitochondrial depolarization, caspase activation and mixed apoptotic/necrotic cell death [18]. Recent studies have suggested novel mechanisms of radiosensitizer-induced cell death that involve stress by reactive oxygen species (ROS), lysosomal membrane permeabilization (LMP) and autophagy inhibition [19]. Tumor cells may use autophagy as a means of surviving the metabolic stress encountered during radiation or systemic therapy; therefore, autophagy inhibition by CLQ may explain the radiosensitization property of this drug. Autophagy inhibition is a consequence of lysosomotropic effects (the blocking of lysosomal function, acidification and trafficking) and the degradation of autophagosomes, some of which are also effects of CLQ, as described above [16-19,26]. Two antimalarial drugs, CLQ and hydroxychloroquine, inhibited the autophagy induced by p53­mediated apoptosis and augmented the anticancer activity of cyclophosphamide in Myc-driven lymphoma [27,28]. The inhibition of autophagy by chloroquine increased cell death in imatinib-resistant, BCR­ABL­positive CML cell lines and enhanced the effect of the HDAC inhibitor vorinostat [29-31].Chloroquine and the antimalarial drug quinacrine sensitized gastrointestinal stromal tumor cells toward treatment with imatinib both in vitro and in vivo [32]. Eventhough that chloroquine demonstrated in experimental models potential synergistic effect with radiation therapy, response rates in our study were not superior to placebo. Chloroquine could increase necrosis in metastatic lesions, inhibiting autophagy [16-19,26] and no downsize brain metastases according with RECIST criteria, caused a lack of response in conventional imaging methods such as MRI.

In our trial, CLQ did not increase WBI toxicity and did not cause any visual effects when it was administered concurrently with WBI or after WBI. This result is in contrast to findings that other radiosensitizers may cause hematological and gastrointestinal toxicity [11,33].

Many clinical trials of radiosensitizers, including ionidamine, metronidazole, misonodazole, motexafin gadolinium, BUdr, efaproxiral and thalidomide, have had the goal of improving the outcomes of patients with BM, however, the results of these studies are controversial, and no significant improvements in OS or PFS have been shown. Viani et al. [34] recently conducted a meta-analysis of 8 RCTs with 2317 patients. The results demonstrated that WBI combined with radiosensitizers did not produce a meaningful improvement in overall survival compared with WBI alone (OR = 1.03, 95% CI0.84-1.25, p = 0.77).

Previous phase II trials have also suggested that temozolomide improves the local control of BM without increasing toxicity (except lymphopenia) [12]. Potential benefits of CLQ use instead of temozolomide for patients with BM are the lower price and favorable safety profile of CLQ. CLQ is a widely studied drug with a well-established toxicity profile and a low risk of potential side effects, except when used at higher doses and for longer periods to treat malaria and autoimmune diseases. The classical ocular toxicity (retinopathy and macular degeneration) associated with CLQ treatment is not observed with the doses of CLQ used to treat BM or GBM.

Our study had methodological limitations, including a small sample size and the absence of neurocognitive evaluations, which may limit our results. In conclusion, treatment with CLQ concurrently with radiotherapy for two weeks and alone for two weeks thereafter was well tolerated and suggested an improved BMPFS .These results require confirmation in a phase 3 trial.

## Competing interests

The authors declare that they have no competing interests.

## Authors’ contributions

Conception and design: OA, AC and CA-S. Financial support: OA. Provision of study materials or patients: LR-P, MG-P, AC, CG, RN-G, CA and OA. Data analysis and interpretation: LR-P, MG-P, AC, AO-G, CG,YD-G and OA. Manuscript preparation: LR-P, MG-P, AC, AO-G, CG, RN-G, YD-G, CA and OA. Final approval of the manuscript: LR-P, MG-P, AC, AO-G, CG, RN-G, YD-G, CA and OA.

## Supplementary Material

Additional file 1 Relationship between objective response, clinical factors and treatment. Click here for file

Additional file 2 Adverse Events. Click here for file

Additional file 3 Factors Associated with Event Free Survival in patients with Brain Metastases. Click here for file

Additional file 4 EORTC QLQ-C30 Scores. Click here for file

Additional file 5 Quality of life. Click here for file
