# Supplementary material for: Phase II randomized, double-blind, placebo-controlled study of whole-brain irradiation with concomitant chloroquine for brain metastases
Source: Radiat Oncol. 2013 Sep 8;8:209. doi: 10.1186/1748-717X-8-209 (PMC3848663; doi:10.1186/1748-717X-8-209)
Supplement: Additional file 1 — Relationship between objective response, clinical factors and treatment. [file 1748-717X-8-209-S1.docx]

| Additional file 1: Relationship between objective response, clinical factors  and treatment | | | |
| --- | --- | --- | --- |
| Factor | ORR  (%) | Univariate  odds ratio  (95% CI) | *P* |
| Gender |  |  |  |
| Male | 66 | 2.0 (0.425-9.4) | 0.376 |
| Female | 50 |  |  |
|  |  |  |  |
| Age (years) |  |  |  |
| <55 | 40.9 | 3.1 (8.6-11.3) | 0.78 |
| ≥55 | 68.4 |  |  |
|  |  |  |  |
| KPS |  |  |  |
| <80 | 60 | 0.7 (0.1-5) | 0.762 |
| ≥80 | 52.8 |  |  |
|  |  |  |  |
| Metastases |  |  |  |
| ≤4 | 51.6 | 1.4 (0.3-5.9) | 0.644 |
| ≥4 | 60 |  |  |
|  |  |  |  |
| Histology |  |  |  |
| NSCLC and others | 50 | 0.57 (0.138-2.4) | 0.43 |
| Breast cancer | 63.6 |  |  |
|  |  |  |  |
| RPA I | 33.3 | 2.4 (0.2 - 29.6) | 0.463 |
| II | 55.3 |  |  |
| Time of brain metastasis  During primary tumor diagnosis  Recurrence or progression | 60  40 | 1.5 (0.4-5.4) | 0.536 |
| Treatment |  |  |  |
| Control arm | 52.6 | 1.08 (0.3 - 3.7) | 0.92 |
| CLQ arm | 54.5 |  |  |
|  |  |  |  |
| Abbreviations: CLQ, chloroquine ; KPS, Karnofsky performance score; NSCLC, non-small-cell lung cancer; RPA, recursive partitioning analysis; CHT, chemotherapy | | | |
