# Supplementary material for: Phase II randomized, double-blind, placebo-controlled study of whole-brain irradiation with concomitant chloroquine for brain metastases
Source: Radiat Oncol. 2013 Sep 8;8:209. doi: 10.1186/1748-717X-8-209 (PMC3848663; doi:10.1186/1748-717X-8-209)
Supplement: Additional file 2 — Adverse Events. [file 1748-717X-8-209-S2.docx]

**Additional File 2:** Adverse Events

| TOXICITY | CLQ arm (n=39) | | | Control arm (n=34) | | |
| --- | --- | --- | --- | --- | --- | --- |
|  | Grade 1 | Grade 2 | Grade 3 | Grade 1 | Grade 2 | Grade 3 |
| Clinical | | | | | | |
| Headache | 15 | 3 | 0 | 16 | 6 | 2 |
| Seizures | 0 | 0 | 0 | 3 | 0 | 0 |
| BlurryVision | 2 | 0 | 0 | 4 | 0 | 0 |
| Drowsiness | 11 | 0 | 0 | 7 | 0 | 2 |
| Nausea | 16 | 4 | 1 | 11 | 2 | 0 |
| Vomit | 11 | 1 | 0 | 2 | 2 | 1 |
| Hyporexia | 7 | 4 | 0 | 3 | 1 | 0 |
| Constipation | 1 | 0 | 0 | 1 | 1 | 0 |
| Fatige | 6 | 4 | 1 | 5 | 6 | 0 |
| Hipoacusia | 0 | 0 | 0 | 2 | 0 | 0 |
| Tinnitus | 0 | 0 | 0 | 2 | 0 | 0 |
| Laboratories | | | | | | |
| Hb | 3 | 3 | 0 | 4 | 1 | 0 |
| Leucocites | 1 | 0 | 0 | 5 | 0 | 1 |
| Plaquets | 1 | 0 | 0 | 2 | 1 | 0 |
| Albumine | 19 | 5 | 2 | 12 | 9 | 0 |
| ASAT | 4 | 0 | 0 | 2 | 1 | 0 |
| ALAT | 4 | 1 | 0 | 1 | 2 | 0 |
| DHL | 10 | 1 | 1 | 6 | 0 | 0 |

Abbreviations: CLQ: Choloroquine; Hb: Hemoglobine; ASAT: aspartate aminotransferase;

ALAT: alanine aminotransferase; DHL: Lactate dehydrogenase
