# Supplementary material for: Phase II randomized, double-blind, placebo-controlled study of whole-brain irradiation with concomitant chloroquine for brain metastases
Source: Radiat Oncol. 2013 Sep 8;8:209. doi: 10.1186/1748-717X-8-209 (PMC3848663; doi:10.1186/1748-717X-8-209)
Supplement: Additional file 3 — Factors Associated with Event Free Survival in patients with Brain Metastases. [file 1748-717X-8-209-S3.docx]

| Additional File 3: Factors Associated with Event Free Survival in patients with Brain Metastases | | | |
| --- | --- | --- | --- |
| Factor | Median  (months) | CI (95%) | UnivariateAnalysis  p |
|  |  |  |  |
| Gender |  |  |  |
| Masculine | 6.9 | (5.9 - 8.1) | 0.163 |
| Femenine | 7.5 | (3.7-10.5) |  |
|  |  |  |  |
| Age |  |  |  |
| <55 | 6.9 | (5 - 88) | 0.522 |
| ≥55 | 8.4 | (5.3 -11.6) |  |
|  |  |  |  |
| KPS |  |  |  |
| <80 | 4.6 | (1.3 – 7.8) | 0.19 |
| ≥80 | 8.4 | (4.9 -11.9) |  |
|  |  |  |  |
| Number of metastases |  |  |  |
| <4 | 10.1 | (7.1 - 13.0) | 0.004 |
| ≥4 | 2.9 | (1.1 - 4.8) |  |
|  |  |  |  |
| Histology |  |  |  |
| NSCLC and others | 6.9 | (4.1 - 9.9) | 0.862 |
| Breast | 7.5 | (4.6 -10.3) |  |
|  |  |  |  |
| Time of brain metastasis |  |  |  |
| During primary tumor diagnosis | 7.88 | (5.4-12.3) | 0.675 |
| During Recurrence | 6.7 | (4.89 - 8.5) |  |
|  |  |  |  |
| Treatment |  |  |  |
| Control arm | 7.4 | (6.1 - 8.8) | 0.126 |
| CLQ arm | 7.5 | (3.1 - 11.8) |  |
|  | | | |
| Abbreviations: Event Free Survival: PFS and/or Death. SE,Standard error; KPS, Karnofsky performance status; NSCLC Non small cell lung cancer; CHT, Chemotherapy; CLQ, cloroquine; ^§^*P* log-rank test. | | | |
