# Supplementary material for: Phase II randomized, double-blind, placebo-controlled study of whole-brain irradiation with concomitant chloroquine for brain metastases
Source: Radiat Oncol. 2013 Sep 8;8:209. doi: 10.1186/1748-717X-8-209 (PMC3848663; doi:10.1186/1748-717X-8-209)
Supplement: Additional file 4 — EORTC QLQ-C30 Scores. [file 1748-717X-8-209-S4.pptx]

## Slide 1
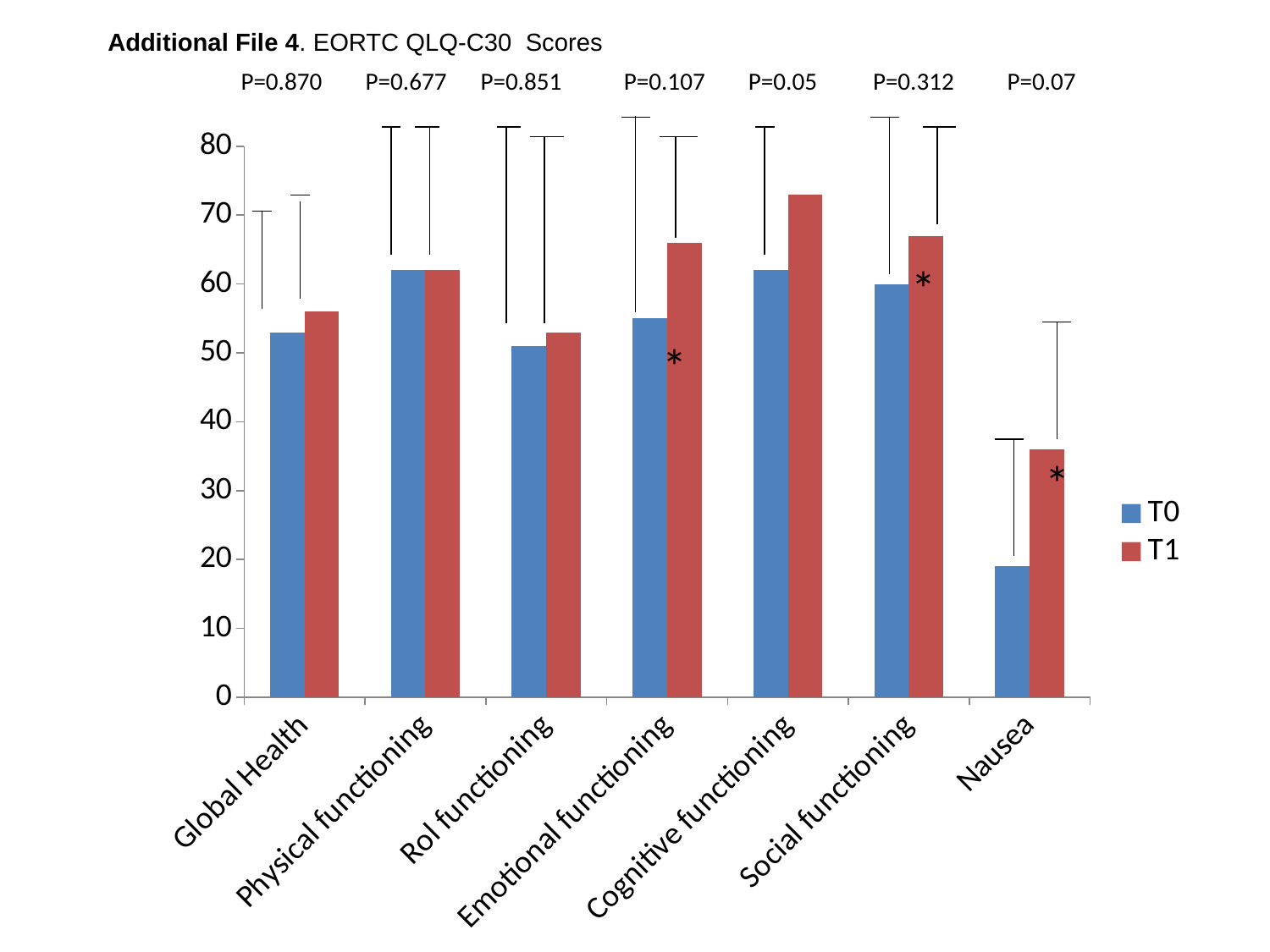

Additional File 4. EORTC QLQ-C30 Scores
P=0.851
P=0.870
P=0.677
P=0.107
P=0.05
P=0.312
P=0.07
### Chart
| Category | T0 | T1 |
|---|---|---|
| Global Health | 53.0 | 56.0 |
| Physical functioning | 62.0 | 62.0 |
| Rol functioning | 51.0 | 53.0 |
| Emotional functioning | 55.0 | 66.0 |
| Cognitive functioning | 62.0 | 73.0 |
| Social functioning | 60.0 | 67.0 |
| Nausea | 19.0 | 36.0 |
