# Supplementary material for: Phase II randomized, double-blind, placebo-controlled study of whole-brain irradiation with concomitant chloroquine for brain metastases
Source: Radiat Oncol. 2013 Sep 8;8:209. doi: 10.1186/1748-717X-8-209 (PMC3848663; doi:10.1186/1748-717X-8-209)
Supplement: Additional file 5 — Quality of life. [file 1748-717X-8-209-S5.docx]

| Additional File 5: Quality of life | | | |
| --- | --- | --- | --- |
| Factor | Control arm | CLQ arm | Univariate Analysis  p |
|  |  |  |  |
|  |  |  |  |
| Global Health | 5.9 | -5.5 | 0.195 |
|  |  |  |  |
| Physical  Status | 0.31 | -4.1 | 0.616 |
|  |  |  |  |
| Functional  Status | 7.14 | -6.3 | 0.331 |
|  |  |  |  |
| Cognitive  Status | 17.15 | 2.4 | 0.132 |
|  |  |  |  |
| Emotional  Status | 18.3 | 1.2 | 0.153 |
|  |  |  |  |
| Social  Status | 12.7 | -1.6 | 0.164 |

Abbreviations: CLQ, chloroquine
